# The application of extended reality technology-assisted intraoperative navigation in orthopedic surgery

**DOI:** 10.3389/fsurg.2024.1336703

**Published:** 2024-02-05

**Authors:** Dongxiao Bian, Zhipeng Lin, Hao Lu, Qunjie Zhong, Kaifeng Wang, Xiaodong Tang, Jie Zang

**Affiliations:** ^1^ Department of Musculoskeletal Tumor, Peking University People’s Hospital, Beijing, China; ^2^ State Key Laboratory of Virtual Reality Technology and Systems, Beihang University, Beijing, China; ^3^ Traumatic Orthopedic Department, Peking University People’s Hospital, Beijing, China; ^4^ Arthritis Clinic and Research Center, Peking University People’s Hospital, Beijing, China; ^5^ Spinal Surgery Department, Peking University People’s Hospital, Beijing, China

**Keywords:** extended reality (XR), mixed reality (MR), virtual reality (VR), augmented reality (AR), intraoperative navigation, orthopedic surgery

## Abstract

Extended reality (XR) technology refers to any situation where real-world objects are enhanced with computer technology, including virtual reality, augmented reality, and mixed reality. Augmented reality and mixed reality technologies have been widely applied in orthopedic clinical practice, including in teaching, preoperative planning, intraoperative navigation, and surgical outcome evaluation. The primary goal of this narrative review is to summarize the effectiveness and superiority of XR-technology-assisted intraoperative navigation in the fields of trauma, joint, spine, and bone tumor surgery, as well as to discuss the current shortcomings in intraoperative navigation applications. We reviewed titles of more than 200 studies obtained from PubMed with the following search terms: extended reality, mixed reality, augmented reality, virtual reality, intraoperative navigation, and orthopedic surgery; of those 200 studies, 69 related papers were selected for abstract review. Finally, the full text of 55 studies was analyzed and reviewed. They were classified into four groups—trauma, joint, spine, and bone tumor surgery—according to their content. Most of studies that we reviewed showed that XR-technology-assisted intraoperative navigation can effectively improve the accuracy of implant placement, such as that of screws and prostheses, reduce postoperative complications caused by inaccurate implantation, facilitate the achievement of tumor-free surgical margins, shorten the surgical duration, reduce radiation exposure for patients and surgeons, minimize further damage caused by the need for visual exposure during surgery, and provide richer and more efficient intraoperative communication, thereby facilitating academic exchange, medical assistance, and the implementation of remote healthcare.

## 1 Introduction

Extended reality (XR) technology refers to any situation where real-world objects are enhanced with computer technology, including virtual reality (VR), augmented reality (AR), and mixed reality (MR). It provides users with an immersive experience by integrating or manipulating computer-generated digital content (1). The term “virtual reality” was coined as early as 1987. The definitions of VR and AR were first elaborated by Milgram and Kishino in their description of the “virtual continuum.” The definition of VR proposed by Jonathan Steuer is considered more comprehensive: “virtual reality is defined as a real or simulated environment in which a perceiver experiences telepresence” (2). In virtual reality, users are fully immersed in a simulated digital environment and disconnected from the real world. The subject is placed in a well-developed interactive virtual environment and interacts with it by using multiple sensors and controllers to create a sense of presence. In augmented reality technology, virtual images and objects are overlaid onto the real environment, providing enhancements of the user's experience. In 1994, Paul Milgram and Fumio Kishino first defined MR as “the merging of real and virtual worlds to produce new environments and visualizations. Virtual and real objects coexist, and users can interact with both in real time” (1).

The earliest application of virtual reality in practical fields was in induction training for the US Air Force, which earned Tom Furness the title of “Father of Virtual Reality” (3). Since then, VR has flourished. In the field of clinical medicine, VR was first introduced by Robert Mann into orthopedics in the 1980s (4). Subsequently, in 1998, head-mounted virtual reality devices were used for the treatment of arachnophobia in pathological therapy (5).

In this review, we aimed to summarize the current application of XR-technology-assisted intraoperative navigation in orthopedic surgery, as well as to provide a detailed discussion of its effectiveness and superiority. Additionally, we discussed the limitations of the application of XR technology from the perspective of both the technology itself and the evaluation and validation metrics.

## 2 XR technology in orthopedic trauma surgery

### 2.1 The application of VR and AR technologies in minimally invasive surgery for pelvic fractures

The repair of pelvic fractures emphasizes the maintenance of the anatomical shape of the pelvis and the restoration of its biomechanical characteristics. In the reconstruction of acetabular fractures, the principles of anatomical reconstruction and stable fixation of the articular surface should be followed. However, due to the complexity of the pelvic structure, achieving this in actual surgery is challenging and requires the surgeon to have extensive clinical experience to successfully complete it. The effectiveness of using VR for path guidance in percutaneous screw fixation in the sacroiliac joint was evaluated by Tonetti et al. They assessed the accuracy of 23 surgeons in inserting guide wires according to a predetermined procedure during cadaver experiments and found that VR simulation could reduce the need for intraoperative fluoroscopy during the placement of the guide wire in cadavers. Novice surgeons who had good anatomical knowledge of the lumbosacral joint but lacked surgical proficiency benefited the most from this VR guidance (6).

Wang et al. developed a novel AR-based sacroiliac screw placement navigation system for preoperative planning and evaluated its feasibility and accuracy in cadaver experiments. Six complete pelvic specimens were scanned by using CT imaging, and the pelvis and blood vessels were segmented into 3D models. Based on this, the ideal trajectory for sacroiliac screw placement was designed and visualized as a cylinder. A virtual 3D model was overlaid on the surgeon's field of view while using a head-mounted display (HMD) for assistance, and the screw was fixed according to the trajectory represented by the cylinder. The method was proven to be feasible, accurate, and able to serve as an aid in percutaneous sacroiliac screw implantation surgery (7).

The conventional surgical treatment method for combined pelvic and acetabular fractures requires the complete exposure of the fracture site and the implant contour after fracture reduction during surgery in order to adapt the reconstruction plate to the narrowing of the pelvis. This requirement for exposure often leads to prolonged surgical time and significant damage and bleeding (8).

Shen et al. developed a specific AR-assisted preoperative reconstruction plate design system for unilateral pelvic and acetabular fracture reduction and internal fixation surgery. This system helped simulate fracture reduction and plate design. Surgeons were able to design the reconstruction plate and its final shape after bending and to formulate the surgical plan for its placement. By using this technology for personalized preoperative surgical planning, the intra-operative implant templating procedure was omitted, thus minimizing surgical trauma while achieving satisfactory reduction and fixation (9).

### 2.2 The application of AR and MR technologies in spinal fractures

Auloge et al. investigated the safety and feasibility of AR technologies in combination with artificial intelligence (AI) software (AR/AI technology) in percutaneous vertebral body augmentation surgery. This prospective randomized study included 10 patients with vertebral compression fractures who underwent the surgery. The use of AR/AI technology and traditional surgical methods were compared in terms of the accuracy of needle placement, the duration of the surgery, and complications. In all cases, the AI software successfully identified the structures and generated a safe and accurate trajectory. Although the time required for needle placement using AR/AI technology was longer than that using standard fluoroscopic guidance, the accuracy of both methods was similar. The radiation dose–area product (182.6 ± 106.7 mGy cm^2^, 5.2 ± 2.6 s) in the AR/AI group was significantly lower than that in the standard fluoroscopic guidance group (367.8 ± 184.7 mGy cm^2^, 10.4 ± 4.1 s), reducing the radiation exposure of both patients and operators during the procedure (10).

Li et al. investigated the feasibility of using MR technology for the treatment of lumbar vertebral fractures. In seven cases of posterior lumbar surgery, an MR-based three-dimensional image navigation system (MITINS) was used to assist in the placement of pedicle screws. Postoperative x-ray images were taken to evaluate the feasibility and safety of pedicle screw placement. A total of 57 pedicle screws were safely and accurately implanted into a three-dimensional model of the lumbar spine by using the MITINS technology. The application of MITINS did not require additional positioning information from x-ray images. Postoperatively, patients experienced a reduction in pain scores and the disability index. This study demonstrated that the application of MITINS in lumbar vertebral fracture surgery is feasible, safe, and accurate (11).

**Figure 1:**
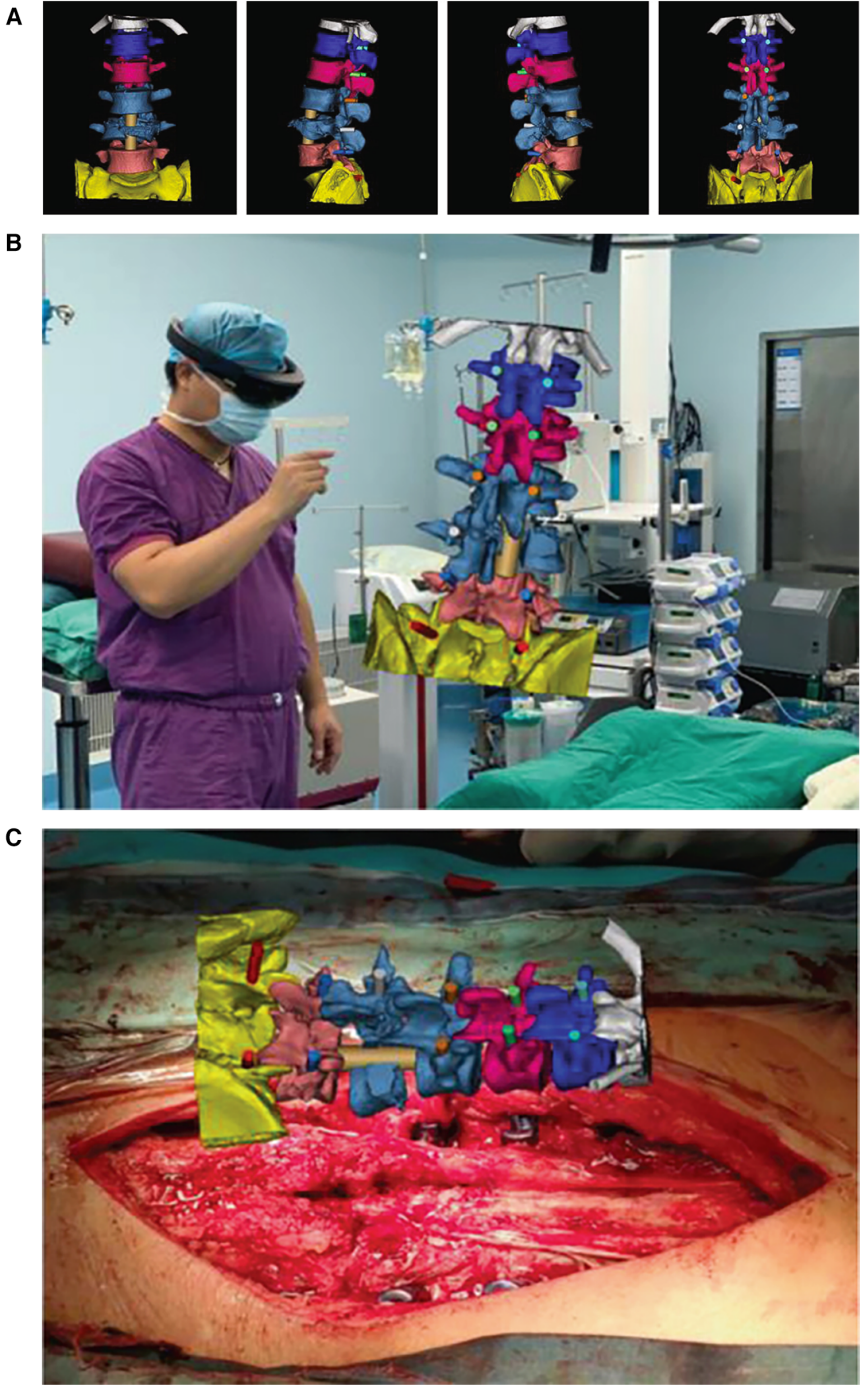
The spinal surgery in this case was performed with the assistance of MITINS. (**A**) Preoperative surgical demonstration. (**B**,**C**) The application of MITINS in LF (lumbar fusion) surgery (11).

Figure 1 shows how the spinal surgery was performed with the assistance of MITINS in this case.

### 2.3 The application of AR technology in scapular fractures

Guo et al. evaluated the feasibility, accuracy, and effectiveness of using AR technology and a three-dimensional (3D)-plate-library-assisted minimally invasive posterior surgery (referred to as the scapular AR system) for the treatment of scapular fractures. By employing this strategy, the surgical time was significantly shorter than that in the conventional surgery group (−28.75 min), and the intraoperative blood loss was significantly less than that of the conventional surgery group (−81.94 ml). No patients had intraoperative or postoperative complications (12).

### 2.4 The application of AR and VR technologies in joint fractures

Zemirline et al. designed an AR-based navigation system that combined with wrist arthroscopy to provide clear visualization of wrist joint fractures, positional relationships, and displacements. This system accurately displayed the fracture location and instrument position in the same field of view during complex wrist joint fracture surgeries, resulting in a significantly higher success rate than that of traditional surgical methods (13).

To accurately assess bone displacement during joint movement, Jeung et al. developed a real-time AR surgical guidance system for wrist arthroscopy based on *in vivo* CT imaging of bone displacement models. This system was designed to address errors caused by wrist joint traction. It enabled the visualization of hidden bones and expanded the limited field of view in arthroscopy to display the precise positions of wrist bones during joint movement. It is worth mentioning that the proposed bone displacement compensation can also be applied to other joints (14).

The applications of XR-assisted intraoperative navigation in trauma surgery are shown in Table 1.

**Table 1:** The application of XR-assisted intraoperative navigation in trauma surgery.

Navigation system	Author	Year	VR/AR/ MR	Image	Application	Outcomes
N/A (Navigation stimulator)	Tonetti et al. (6)	2009	VR	x-ray	T	A 2D imaging system simulator that can be applied to train novice doctors in performing operations in a real 3D environment.
N/A	Wang et al. (7)	2016	AR	CT	P	A novel sacroiliac screw placement navigation system based on (AR) was designed for preoperative planning, and its feasibility and accuracy were demonstrated.
N/A	Shen et al. (9)	2013	AR	CT	P	A personalized AR-assisted preoperative reconstruction and plate design system was developed for unilateral pelvic and acetabular fracture reduction and internal fixation surgery. Its feasibility was demonstrated, in addition to its enhancement of efficiency.
AR/AI-guided	Auloge et al. (10)	2020	AR	CT	I	The feasibility of using novel navigation tools based on AR and AI for surgical treatment of vertebral compression fractures was discussed. The results suggested that these tools can help reduce patients’ radiation exposure.
MITINS	Li et al. (11)	2021	MR	CT	P/I	An MR-based intraoperative three-dimensional image navigation system (MITINS) was proposed, and it was also used for preoperative reconstruction and plate design.
AR-scapular system	Guo et al. (12)	2022	AR	CT	I	The feasibility, accuracy, and effectiveness of using AR and a three-dimensional (3D) plate library for assisted minimally invasive posterior surgery (i.e., a scapula AR system) in the treatment of scapular fractures were evaluated.
N/A	Zemirline et al. (13)	2013	AR	N/A	I	A navigation system based on electromagnetic sensors was developed, and its accuracy in video-assisted surgery was evaluated. The results showed that the use of AR could shorten the surgical time and improve the surgeon's attention.
N/A	Jeung et al. (14)	2023	AR	CT	I	A real-time (AR) surgical guidance system was designed specifically for wrist arthroscopy procedures to address errors caused by wrist joint distraction.

AR, augmented reality; VR, virtual reality; MR, mixed reality; N/A, not applicable; CT, computed tomography; T, training; P, preoperative; I, intraoperative.

## 3 XR technology in joint surgery

### 3.1 The application of MR technology in shoulder joint surgery

Berhouet and colleagues used a new method in 2019 to simulate the pre-disease anatomy of the glenoid and project it onto the surgical field to assist in the placement of glenoid components, allowing surgeons to better understand the pre-disease anatomy for the accurate placement of glenoid guide wires (15).

Kriechling and colleagues created a customized positioning device for accurately positioning the scapula, the coracoid process, and the glenoid by aligning the intraoperative surface with 3D CT. After intraoperative registration, the 3D scapula image and planned guide wire trajectory were projected onto the surgeon's field of view through holographic glasses. Postoperative CT assessments showed a higher accuracy of the guide wire trajectory when using this MR technology. The 3D modeling method resulted in an average trajectory error (including the version angle and inclination angle) of 2.7° ± 1.3° and an entry point error of 2.3 ± 1.1 mm, while the conventional method had an average trajectory error of 3.8 ± 1.7 and an entry point error of 3.5 ± 1.7 mm (16, 17).

Schlueter-Brust et al. and Gregory et al. further investigated the accuracy of shoulder joint guide pin placement without intraoperative registration by using the preoperative planning of three-dimensional scapula images that were overlaid with an MR device. Their results showed an average trajectory error (including the version angle and inclination angle) of 3.9° ± 2.4° and an entry point error of 2.4 ± 0.7 mm. Gregory and colleagues also performed reverse total shoulder arthroplasty (rTSA) using HoloLens2. During the surgery, HoloLens2 projected the preoperative planning directly onto the patient's joint without the need for intraoperative registration. At the same time, it enabled video conferencing with four other surgeons from different countries, who could provide real-time advice and adjust the heads-up display (HUD). Although there were no actual measurements of the acetabular component's position, the authors suggested that it was appropriate, and the surgery time was only 90 min.

**Figure 2:**
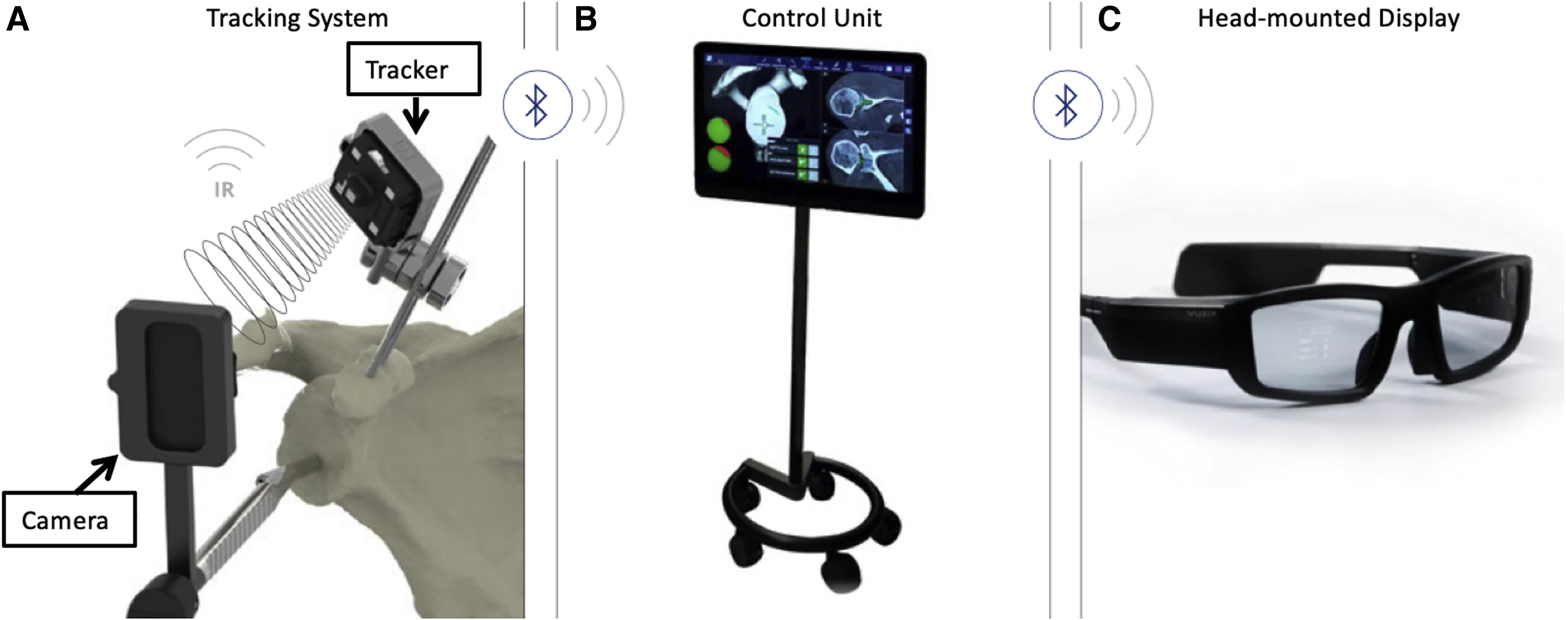
(**A**) Tracking system (TS) for the real-time tracking of an instrument's position relative to anatomical structures by using infrared (IR) disposable sensors (trackers and cameras). (**B**) The control unit (CU) received information from the TS via Bluetooth and integrated this information with the planning. (**C**) The head-mounted display received information from the CU via Bluetooth. The overlay of surgical operations onto the visualized surgical area allowed surgeons to focus on the patient (18).

**Figure 3:**
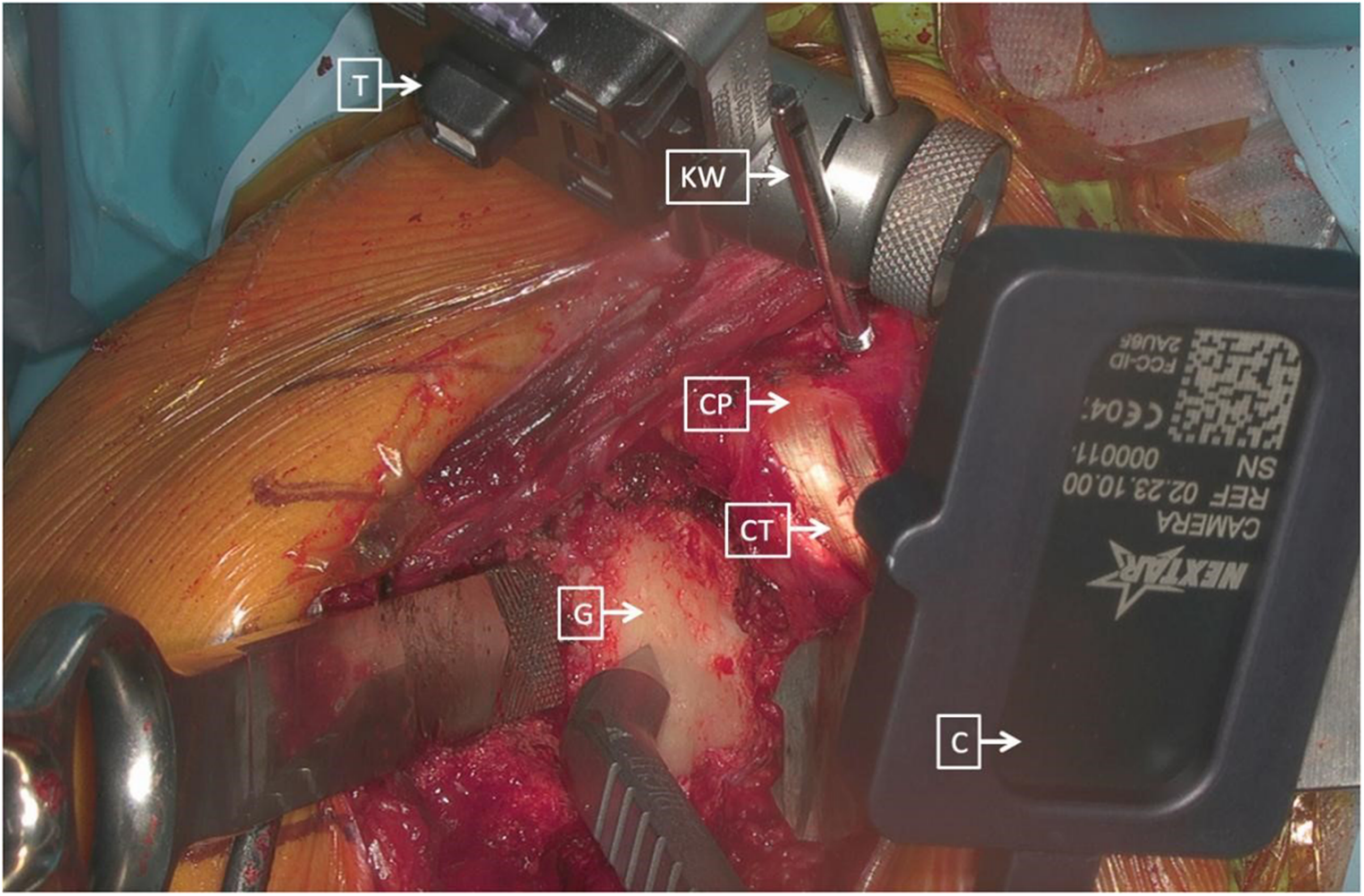
Right shoulder, beach chair position. The tracker (T) was placed and secured on the K-wire (KW) and on the beak (CP), consistently with the cameras (C) that were placed on different instruments (CT, combined with tendons; G, glenoid) (18).

**Figure 4:**
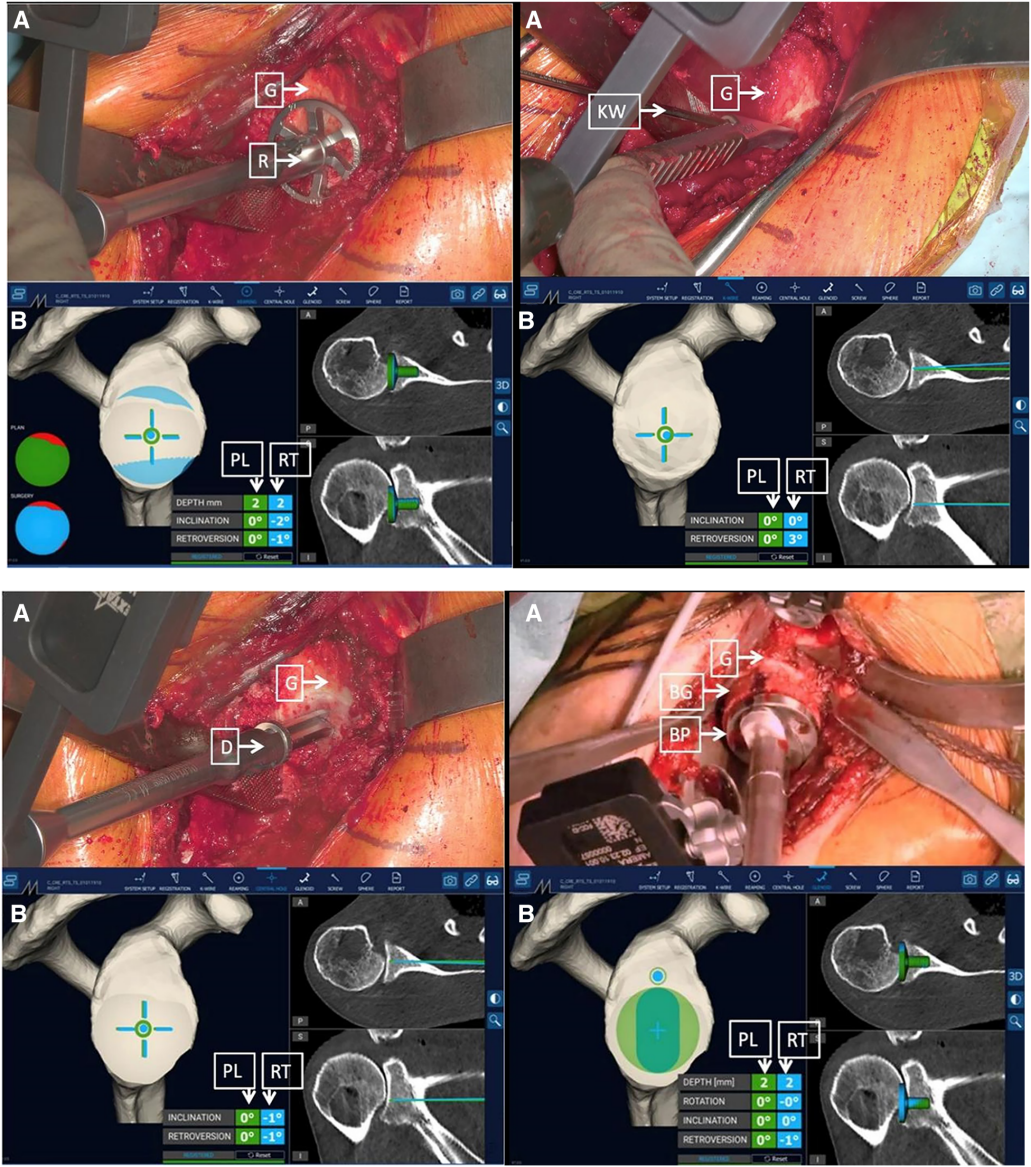
Surgeon's intraoperative field of view. The augmented reality navigation system provided planned (PL) and real-time (RT) pitch and yaw values. On the right side of the image, the two-dimensional CT scan images display the axial (top) and coronal (bottom) PL (green line) and RT (blue line) directions (D, drill; G, glenoid) (18).

Rojas and colleagues described a method that combined MR technology with intraoperative navigation and reported a case of reverse total shoulder arthroplasty (rTSA) while using this method, as shown in Figures 2–4. Throughout all of the steps of the placement of the acetabular component, using a variable-application camera tracker, real-time information regarding the planned trajectory, including the retroversion angle, inclination angle, entry point position, reaming depth, and ideal trajectory for guide and drill placement, was provided to the surgeons through MR technology (18).

### 3.2 The application of MR technology in hip joint surgery

Jevan et al. developed a prototype system script that utilized augmented reality (AR) technology to accurately place components of hip joint implants in preoperatively planned positions. The average absolute deviations (ranges) between the target and actual positions of the acetabulum were 2.9° (−8.7° to 3.3°), 3.0° (−5.7° to 7°), and 1.6 mm (−1.2 to 3.5 mm). In addition, 66% of the results fell within the range of ±5° from the preoperative target orientation (19).

For core decompression surgery on the femoral head, Wang et al. developed an intraoperative navigation system based on augmented reality (AR) technology. This surgical system could visually display the anatomical structures of the surgical area and present preoperative images and virtual guide needles in real time within the intraoperative video. With the guidance of the navigation system, surgeons could accurately insert Kirschner wires into the target lesion area, minimizing intraoperative damage to the maximum extent. Compared with traditional methods, this approach significantly improved the efficiency of positioning and ensured the accuracy of the puncture (20).

Similarly, in arthroscopic hip surgery, Song et al. proposed an augmented reality system for assisting in the placement of the arthroscope during the examination process. Compared with arthroscope placement without additional positioning support, this system allowed for faster and better-angled entry into the surgical area, reducing patient trauma and minimizing x-ray exposure during surgery (21).

### 3.3 The application of MR technology in knee joint surgery

Chen et al. proposed an in-situ augmented reality navigation system based on knee arthroscopy. The system's images accurately reflected the structural information of the joint, with an average error of 0.32 mm. Compared with 2D arthroscopic navigation, this augmented reality navigation system reduced the positioning errors by 2.10 mm and 2.70 mm in the knee joint models and *ex vivo* pig knee experiments, respectively (22).

Tsukada et al. developed an AR-based navigation system that overlaid tibial force lines and tibial osteotomy angles onto the surgical field. This navigation system was used in 11 patients undergoing unicompartmental knee arthroplasty. The target angles for each patient were a coronal varus of 0.7° ± 1.0° and a sagittal posterior tilt of 5.3° ± 1.4°. The postoperative x-ray measurements showed a coronal varus of 2.6° ± 1.2° and a sagittal posterior tilt of 4.8° ± 2.5°. The absolute differences between the target and measured angles were 1.9° ± 1.5° in the coronal plane and 2.6° ± 1.2° in the sagittal plane. No patients experienced complications, such as surgical site infection or periprosthetic fracture (23).

From April 2021 to October 2021, Bennet et al. reported a prospective consecutive study that included a total of 20 patients who underwent total knee arthroplasty while using an augmented-reality-assisted navigation system (ARAN). During the surgery, the ARAN was used to measure the positioning of the femoral and tibial cuts in the coronal and sagittal planes, and the final component positions were measured with postoperative CT scans. The absolute differences between the measurements were recorded to determine the accuracy of the ARAN. Two cases were excluded due to segmentation errors, and the remaining 18 cases were analyzed. The mean absolute errors of the ARAN in the coronal plane of the femur, sagittal plane of the femur, coronal plane of the tibia, and sagittal plane of the tibia were 1.4°, 2.0°, 1.1°, and 1.6°, respectively. No outliers were found in the measurements of the femoral or tibial coronal planes (absolute error within 3°). Three outliers were found in the tibial sagittal plane, with all cases showing a smaller tibial slope (3.1°, 3.3°, and 4°). Five outliers were found in the femoral sagittal plane, with all cases showing an overextension of the components (3.1°, 3.2°, 3.2°, 3.4°, and 3.9°). Augmented reality navigation was able to improve the accuracy of bone resection in total knee arthroplasty, especially with a low rate of component misplacement in the coronal plane (24, 25).

Rossi et al. conducted a systematic review with 14 studies associated with the application of AR in total joint arthroplasty that were included in the final analysis. Among them, four studies reported on the application of AR in total knee arthroplasty (TKA), six studies reported on total hip arthroplasty (THA), one study reported on total elbow arthroplasty (TEA), and three studies reported on reverse shoulder arthroplasty (RSA). For THA, AR can increase the accuracy of acetabular component positioning. In terms of TKA, AR may offer reliable accuracy for the coronal, sagittal, and rotational alignment when utilized for tibial and femoral resection. Regarding TEA and RSA, promising results have been achieved at the preclinical level. However, several technical challenges still need to be resolved before widespread clinical use can be realized. All of the results indicated that AR had the potential to replace conventional navigation devices. However, the main challenge in applying this approach in a practical setting would be to identify suitable image processing algorithms for accurately segmenting a target from the surrounding surgical scene for correct registration (26).

The application of XR-technology-assisted intraoperative navigation in joint surgery is shown in Table 2.

**Table 2:** The application of XR-technology-assisted intraoperative navigation in joint surgery.

Navigation system	Author	Year	VR/AR/MR	Image	Application	Outcomes
N/A	Berhouet et al. (15)	2019	AR	CT	I	A novel approach using AR for assisted total shoulder arthroplasty surgery was proposed.
N/A	Kriechling et al. (27)	2021	MR	CT	I	A customized positioning device was created to accurately locate the acromion, coracoid process, and glenoid cavity.
N/A	Schlueter-Brust et al. (17)	2021	AR	CT	I	The accuracy of shoulder joint guide wire placement without an intraoperative registration process was studied by overlaying preoperative planning of three-dimensional scapular images using AR devices.
N/A	Rojas et al. (18)	2022	AR	CT	I	AR technology was used to provide real-time trajectory information that aligned with the planned path during all steps of glenoid prosthesis placement.
N/A	Jevan et al. (19)	2022	AR	N/A	P/I	A prototype system script was developed to use AR technology to accurately place hip joint implant components in preplanned positions.
N/A	Wang et al. (20)	2022	AR	N/A	I	An AR-based intraoperative navigation system for femoral head core decompression surgery was developed.
N/A	Song et al. (21)	2022	AR	N/A	I	An augmented reality system was proposed to assist in the placement of arthroscopic cameras during joint arthroscopy examinations, allowing for better access to the surgical area, reductions in surgical time, the minimization of patient trauma, and reductions in x-ray exposure during surgery.
N/A	Chen et al. (22)	2021	AR	Intraoperative arthroscopic	I	A knee-arthroscopy-based *in situ* AR navigation system was proposed to visualize the structural information of the joint. This system reduced targeting errors, provided global *in situ* information, and provided detailed anatomical information, facilitating minimally invasive knee joint surgeries.
N/A	Tsukada et al. (23)	2022	AR	N/A	I	An AR-based navigation system was developed to present the tibial mechanical axis and tibial resection angle during single-room total knee arthroplasty with a high level of accuracy.
ARAN	Bennett et al. (24)	2023	AR	CT	P/I	The feasibility of using an augmented reality-assisted navigation (ARAN) system for total knee arthroplasty was explored.

AR, augmented reality; MR, mixed reality; N/A, not applicable; CT, computed tomography; I, intraoperative; P, preoperative.

## 4 XR technology in spinal surgery

### 4.1 The application of AR technology in open spinal surgery

There has been a significant amount of research on and application of intraoperative navigation assisted by AR or MR technology in the placement of pedicle screws (28). Some of the literature included in this review did not provide data on the linear tip error (LTE) and angular trajectory error (ATE), but all of the literature included the Gertzbein–Robbins grading scale (GRS) for intraoperative imaging evaluation. Therefore, for studies without LTE and ATE data, the GRS score was used as an evaluation index for the accuracy of pedicle screw placement.

According to the extent of violation of the pedicle cortex by the screw tip, the GRS score is divided into five grades: A–E. Grade A indicates that the screw tip did not breach the pedicle cortex. Grade B indicates that the screw breached the pedicle cortex by 2 mm or less. Grade C indicates a breach of 2–4 mm. Grade D indicates a breach of 4–6 mm. Grade E indicates a breach of over 6 mm or a breach outside the pedicle (29). Only grades A and B are considered satisfactory surgical outcomes, as grades C–E may result in mild to severe postoperative neurological symptoms during follow-up (30).

Bhatt et al. performed 32 posterior spinal surgeries using the xVision Spine AR navigation system, including 19 open surgeries and 13 minimally invasive surgeries (MISs), with a total of 222 screws used. Among the 222 screws, 208 screws were placed while using AR-assisted navigation. Out of the 208 AR-assisted screws, 187 achieved GRS grade A (89.9%), 11 achieved GRS grade B (5.3%), and 6 achieved GRS grade C (2.9%). During the surgery, four screws were considered misplaced and were corrected by using AR-assisted navigation, and all of them achieved GRS grade A after correction (1.9%). The average length of hospital stay for the 32 patients was 4.1 ± 1.6 days, and no intraoperative or postoperative complications occurred during the hospitalization period. At the two-week follow-up, there were three complications among the 32 patients: one case of deep vein thrombosis (DVT) (3.1%) and two cases of pneumonia (6.3%). None of the patients required revision surgery during the two-week follow-up (31).

Harel et al. performed 17 open posterior spine surgeries using the xVision Spine AR navigation system, with a total of 86 screws used. Among the 86 screws, 73 screws achieved GRS grade A (84.9%), 11 screws achieved GRS grade B (12.8%), and 2 screws achieved GRS grade C (2.3%). Patients were followed up until discharge, and no patients experienced symptoms related to nerve root disease or neurologic impairment after the surgery. One patient developed a hematoma at the incision site after the surgery, which improved after adequate drainage, and they had no other complications. The incision hematoma was considered to be caused by the separation of the paraspinal muscles during the procedure and was not related to the use of the navigation system (32).

Judy et al. performed 12 S2AI (S2 Alar–Iliac) surgeries while using the xVision Spine AR navigation system, with a total of 23 screws used. Among the 23 screws, 22 screws (95.6%) achieved GRS grade A or B, with 21 screws (91.3%) being GRS grade A and 1 screw (4.3%) being GRS grade B. One screw (4.3%) achieved GRS grade C. During hospitalization, no patients experienced symptoms of neurological injury (33).

Molina et al. performed surgery on a 78-year-old female who underwent an L4-S1 decompression, pedicle screw placement, and rod fixation for degenerative spine disease. Six pedicle screws were inserted while using the xVison Spine AR navigation system. Intraoperative computed tomography was used for navigation registration and the assessment of implant accuracy and precision. The clinical accuracy was graded by an independent neuroradiologist, which showed that all six pedicle screws that were inserted in this single case achieved GRS grade A, yielding a combined 100% GRS accuracy. The inserted pedicle screws had a mean linear deviation of 2.07 mm (95% CI: 1.62–2.52 mm) and a mean angular deviation of 2.41° (95% CI: 1.57°–3.25°) (34).

### 4.2 The application of XR technology in minimally invasive spinal surgery

Butler et al. performed 164 minimally invasive spine surgeries (MISs) while using the xVision Spine AR navigation system, and a total of 606 screws were used. Among the 606 screws, 603 screws achieved GRS grade A or B (99.51%), and 3 screws were considered misplaced and were corrected during the surgery. The average time from image and surgical field registration to the final placement of each screw was 3 min and 54 s. Analysis of the learning curve showed that the surgical time for early cases was similar to that of cases where the system was used proficiently. In follow-up at 6–24 months, no patients required revision surgery, and no patients experienced symptoms related to nerve root disease or neurologic impairment after the surgery (35).

Peh et al. conducted a study to determine the clinical accuracy of a navigation technology based on augmented reality surgical navigation (ARSN) for minimally invasive thoracic and lumbar pedicle screw instrumentation in comparison with the standard minimally invasive fluoroscopy-guided technique. In four cadavers, a total of 136 pedicle screws were inserted into thoracic and lumbar vertebrae, among which 68 were inserted based on ARSN and the other 68 were inserted while using fluoroscopy. Each thoracic or lumbar vertebrae had one screw inserted based on ARSN on one lateral and one screw inserted based on fluoroscopy on the other lateral. The accuracy was assessed by three independent raters by using postoperative conventional computed tomography. There were 48 screws placed in the thoracic region and 20 screws placed in the lumbar region using ARSN. The overall accuracy (GRS-A and -B) of ARSN was 94.1% (64/68). As planned, 48/20 screws were placed in the thoracic/lumbar regions, respectively, using fluoroscopy, and 88.2% (60/68) were placed accurately (GRS-A and -B) (36).

Huang et al. reported a study of an augmented reality minimally invasive spine surgery (AR-MISS) system utilized in animal experiments. The authors claimed that with the assistance of this system, the puncture procedure could be visually guided in real time, and the anteroposterior and lateral errors of AR-guided punctures were 2.47 ± 0.86 mm and 2.85 ± 1.17 mm, respectively (37).

The applications of XR-technology-assisted intraoperative navigation in spinal surgery are shown in Table 3.

**Table 3:** The applications of XR-technology-assisted intraoperative navigation in spinal surgery.

Navigation system	Author	Year	VR/AR/ MR	Image	Application	Screw number	Outcomes
xVision spine	Bhatt, et al. (31)	2022	AR	N/A	I	218	The report showed an overall accuracy of 97.1% for the placement of 218 AR-guided screws, with no intraoperative or early postoperative complications.
xVision spine	Harel, et al. (32)	2022	AR	CT	I	86	Seventeen surgeries were successfully completed while using the XVS system. Due to technical issues with the intraoperative scanner, two surgeries were unable to be completed. A total of 86 screws were inserted. The accuracy rate of the XVS system was 97.7%.
xVision spine	Judy, et al. (33)	2023	AR	N/A	I	23	The overall accuracy rate of AR-assisted S2AI screw placement was 95.6% (for Grade 0 and Grade 1 screws). AR enabled spine surgeons to place screws more accurately.
xVison spine	Molina, et al. (34)	2021	AR	CT	I	6	The clinical accuracy for six screws (per the GS grading scale) was 100%. Technical precision analysis yielded a mean linear deviation of 2.07 mm and angular deviation of 2.41°.
xVision spine	Butler, et al. (35)	2023	AR	CT	I	606	The first report on the use of AR for minimally invasive placement of pedicle screws in the spine was presented. A total of 164 cases confirmed the effectiveness and safety of screw placement.
ARSN	Peh, et al. (36)	2020	AR	CT	I	136	The overall accuracy of ARSN was 94% compared with an accuracy of 88% for fluoroscopy. Using ARSN, unsafe screws were only observed in the scoliotic spine.
AR-MISS	Huang, et al. (37)	2023	AR	IF	I	N/A	The results of animal experiments showed that the AR-MISS was accurate and applicable.

AR, augmented reality; N/A, not applicable; CT, computed tomography; IF, intraoperative fluoroscopy; I, intraoperative.

## 5 XR technology in orthopedic tumor surgery

As early as 2001, there were reports of VR technology being applied to preoperative prosthesis design and planning for pelvic tumors. Handels et al. introduced the VIRTOPS (Virtual Operation Planning in Orthopedic Surgery) system, which simulated hip joint reconstruction using a semi-pelvic prosthesis and provided support for the personalized design of modular prostheses with strong anatomical adaptability. The VIRTOPS system enabled complete virtual planning and prosthesis reconstruction in hip joint surgery, as well as the optimization of the design and placement of prostheses. It provides a universal platform for the three-dimensional planning and simulation of orthopedic surgery. It can also be used to simulate the implantation of custom-made prostheses and study their compatibility with the pelvis (38).

**Figure 5:**
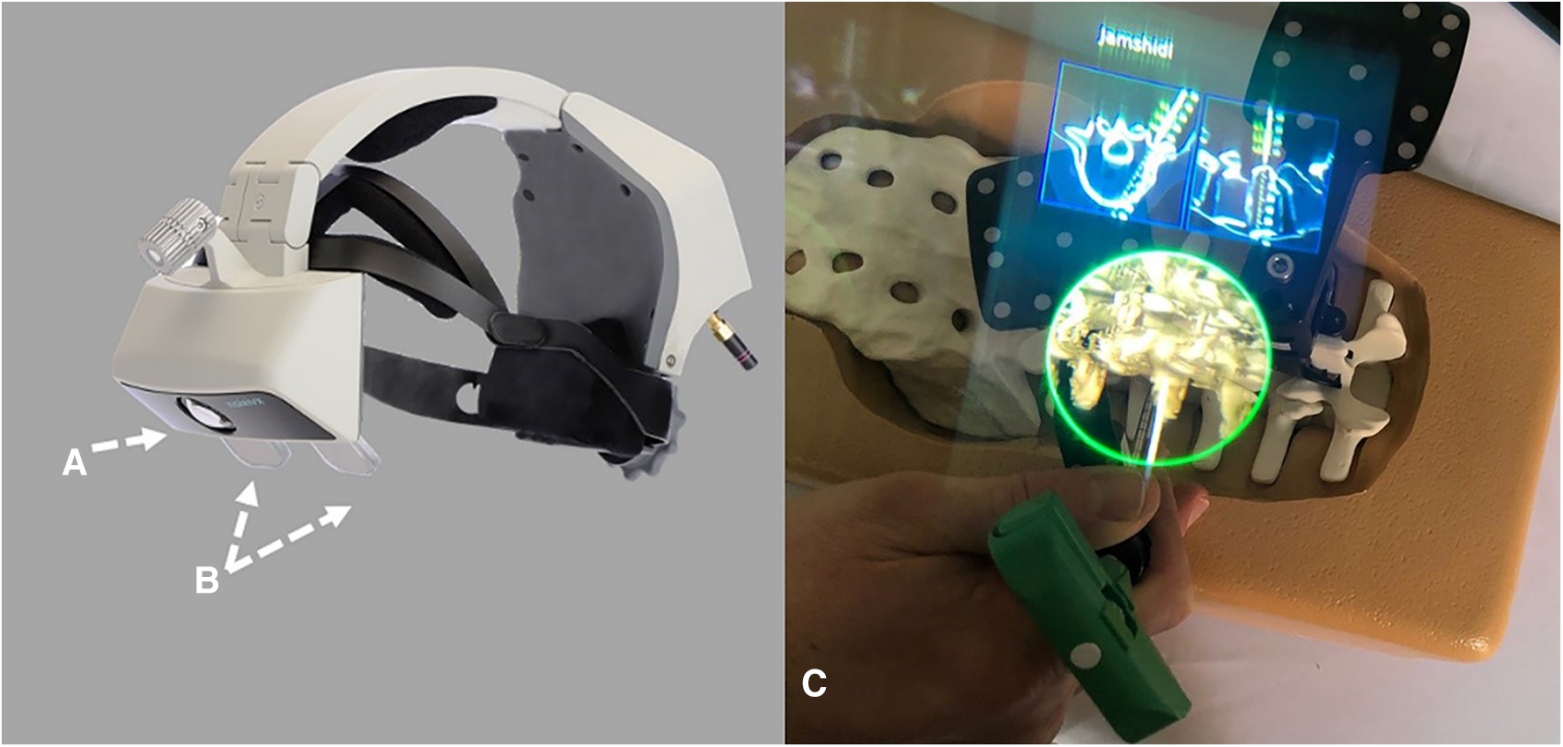
The AR-HMD with ITC (**A**) and a semi-transparent retina display (**B**) can overlay 2D and 3D navigation data with anatomical alignment (**C**) (39).

**Figure 6:**
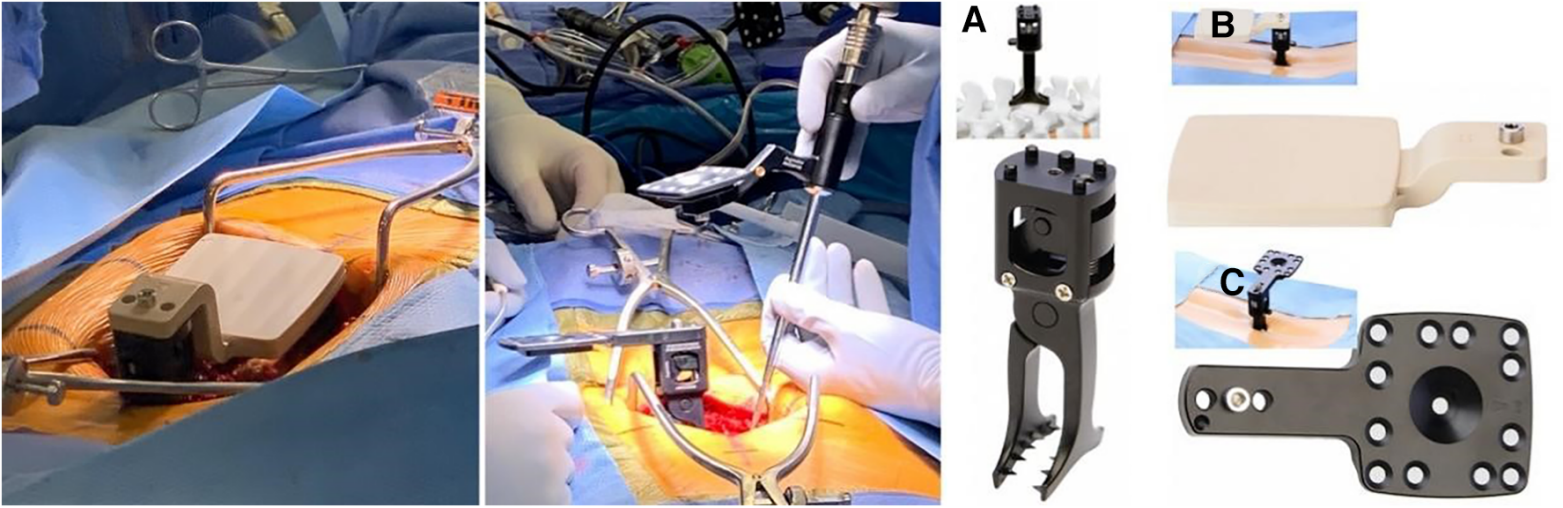
Left: initial registration clamp used for calibration. Middle: The positioning clamp is switched to the working clamp based on the spinous process. Right: The reference clamp for the spinous process (**A**) and the placement of registration markers on the spinous process of L3 (**B**) the registration markers are replaced with reflective navigation markers (**C**), which can be flipped from left to right to minimize line-of-sight interruptions (39).

**Figure 7:**
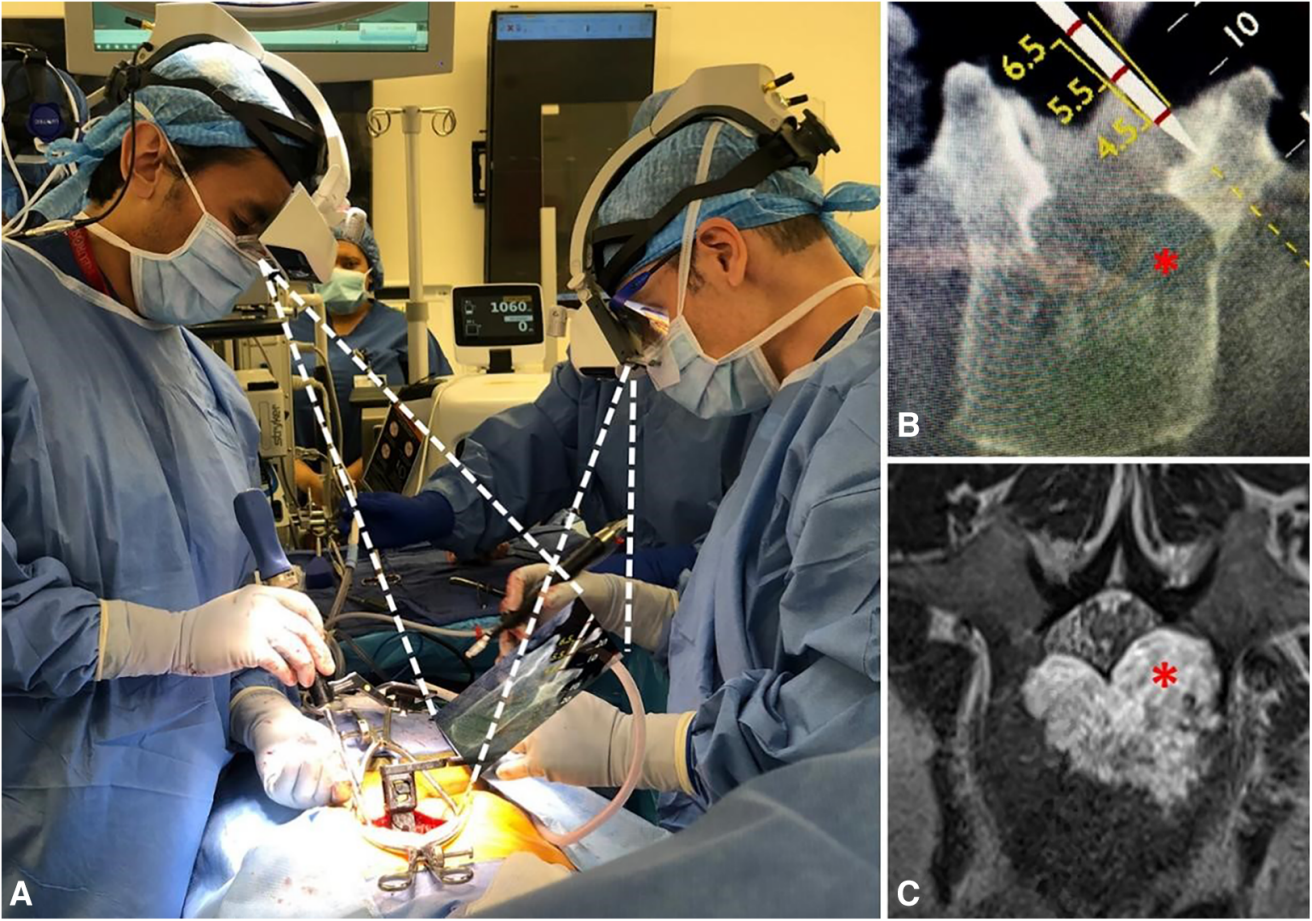
The AR-HMD provides synchronized on-site navigation for two surgeons. The surgeon on the left is using a tracking tool (**A**) to plan an osteotomy (**B**) in order to avoid the extension of a tumor capsule (red asterisk) into the left pedicle and spinal canal (**C**; red asterisk). The surgeon on the right aligns the working tool in parallel with the tracking tool to perform the cutting (39).

**Figure 8:**
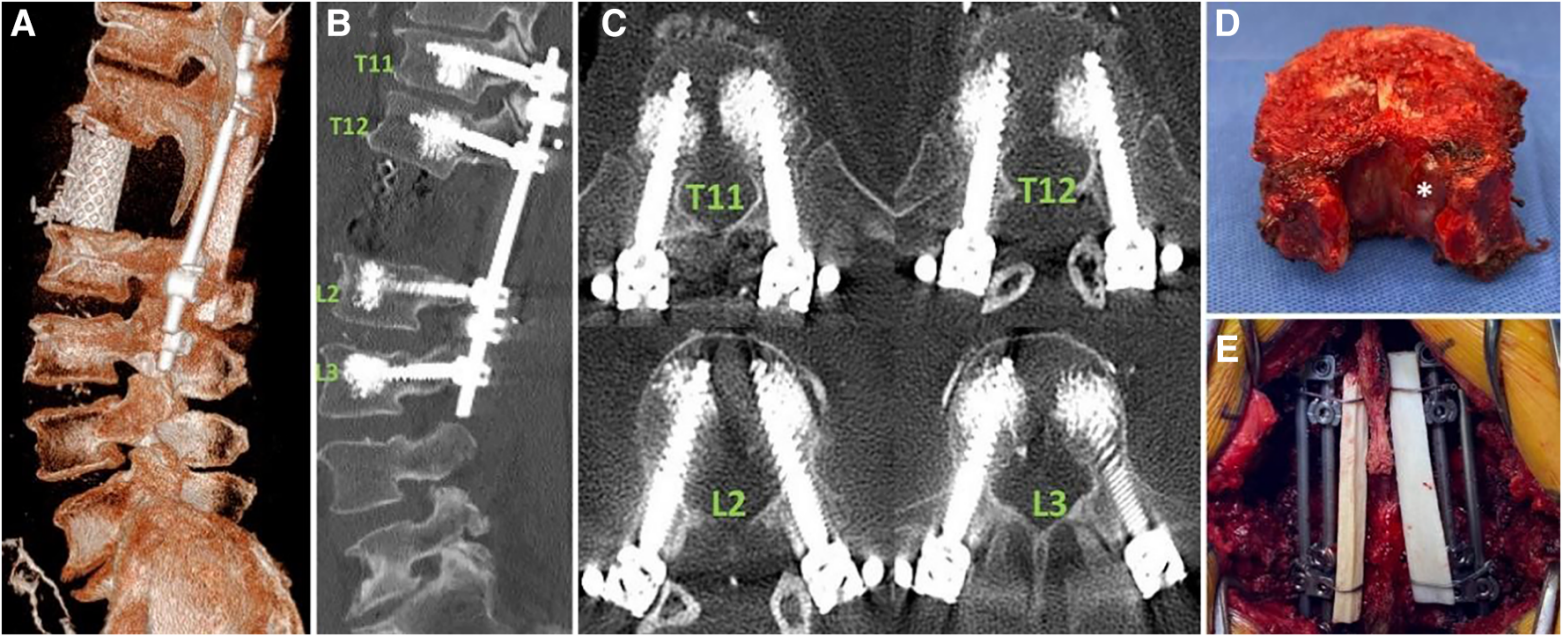
The final spinal reconstruction after the resection. (**A**): The 3D reconstruction demonstrates the placement of the cage. (**B**) and (**C**): The sagittal and axial reconstructions provide evidence that the placement of pedicle screws guided by AR-HMD is correct. (**D**): Whole vertebrectomy specimen. The tumor components within the small tube seen in the MRI are visualized (white asterisk). (**E**): Posterior view of the final construct consisting of four rods and a cable fibular allograft structure (39).

Molina and colleagues reported a case in which ARMSS (augmented-reality-mediated spine surgery) was used as a guidance tool to achieve the complete and extensive resection of an L1 spinal tumor through a posterior approach while avoiding tumor rupture. In this case, they suggested that the use of an AR-HMD (augmented reality head-mounted display) for intraoperative navigation shortened the surgical time, as was expected, and the accuracy of the tumor margin and the placement of pedicle screws was also satisfactory (39). The surgical process with the assistance of ARMSS is shown in Figures 5–8.

Garcia-Sevilla et al. compared the accuracy of custom implant placement for pelvic tumors using traditional methods vs. AR-assisted navigation. They found that the maximum error in implant placement when using traditional methods was up to 54.03 mm, while the maximum error with AR-HMD navigation was only 3.89 mm. Additionally, there was a significant difference in the mean values between the two methods (3.70 mm, 1.40 mm, *P* < 0.005). This study suggested that using AR for intraoperative navigation can significantly reduce errors in implant placement and ensure accurate installation near the target area (40).

The applications of XR-technology-assisted intraoperative navigation in bone tumor surgery are shown in Table 4.

**Table 4:** The application of XR-technology-assisted intraoperative navigation in bone tumor surgery.

Navigation system	Author	Year	VR/AR/ MR	Image	Application	Outcomes
VIRTOPS	Handels et al. (38)	2001	MR	CT	I	Compared to the traditional surgical planning process, it is easier to compare different surgical strategies and their influence on the geometry of a custom-made endoprosthesis. Additionally, the combination of multimodal imaging information (CT and MR) enables precise 3D visualization of intraosseous bone tumors.
ARMSS	Molina et al. (39)	2021	AR	CT/MRI	I	The current AR-HMD platforms can improve the quality of *en bloc* spinal tumor resection surgery, reduce surgical time, and enhance resection accuracy.
N/A	Garcia-Sevilla et al. (40)	2002	AR	CT	I	The results obtained from this study indicate that using AR for guidance can significantly reduce the risk of large placement errors and ensure accurate installation close to the target.

AR, augmented reality; VR, virtual reality; MR, mixed reality; N/A, not applicable; CT, computed tomography; I, intraoperative.

## 6 Discussion

### 6.1 Advantages

This review compiled multiple studies indicating that XR-technology-assisted intraoperative navigation techniques can effectively improve the accuracy of implant placement in the fields of trauma, joint, spine, and bone tumor surgery. As a result, they effectively allow complications caused by the inaccurate placement of implants to be avoided (15–17, 19, 20, 27, 28, 41). In bone tumor surgery, surgeons in the field of orthopedic oncology typically integrate preoperative two-dimensional (2D) images in their minds to assess the three-dimensional (3D) distribution and infiltration of the tumor. They then develop a 3D tumor resection plan—known as osteotomy lines—to achieve a clean surgical margin without tumor infiltration (1). This requires surgeons to have a thorough understanding of anatomical structures, good spatial imagination, and extensive clinical experience. Any slight mistake in surgical planning or execution can result in residual tumor infiltration at the resection margin, increasing the risk of local recurrence and adversely affecting long-term patient survival (42–44). However, as XR provides superior spatial awareness of tumors and reduces the cognitive workload of surgeons, XR-technology-assisted intraoperative navigation techniques can help surgeons obtain a more intuitive visualization of tumors' boundaries (45). As a result, surgeons are more likely to achieve a surgical margin that is free of tumor infiltration.

In addition to improving surgical accuracy, in comparison with traditional navigation techniques, XR-assisted intraoperative navigation techniques can display virtual digital content in holographic images through a head-mounted display (MR HMD). This allows the information to be directly projected onto the retinas of surgeons. The holographic content can include patient data, such as text, clinical images, videos, 2D medical images, processed 3D models, predetermined surgical plans, and CAD-formatted orthopedic implants. The principle of holography is based on the interference of light beams reflected from real objects, as they can better preserve the depth and parallax perception of the original objects without relying on the user's spatial imagination. Users no longer need to rely on printed materials or computer monitors to access medical information but can instead have various patient data presented in a more intuitive manner. The use of XR technology allows surgeons to focus on the surgical field without distractions (46–48) and can present the information and models of the patients that they need in their field of view. Users can interact with holographic images through motion controllers, gestures, voice commands, or their gaze to zoom in, rotate, or move to the appropriate physical space, making such technology easier to use during surgery (1). To summarize, XR-technology-assisted navigation makes it possible for surgeons to acquire an unlimited perspective consisting of the real-time situation of the surgical field, multimodal clinical images, and other processed image data in the operating room. Therefore, despite the lack of strict controls, many studies suggest that XR-assisted intraoperative navigation techniques can shorten surgical time and subsequently reduce radiation exposure for both surgeons and patients (21, 49). Similar viewpoints were proposed in the literature that we reviewed.

In complex orthopedic oncological surgeries, surgeons may consult distant senior doctors or specialists by phone. One obvious advantage of XR technology is that holographic images and the field of view of the primary surgeon can be shared in real time with other users wearing an XR HMD or using computer displays (16), improving their work efficiency and communication. Real-time video of the surgical area from the surgeon's perspective provides richer and more accurate information than traditional audio descriptions through phone calls do. Additionally, remote users can transmit and display key information in the surgeon's line of sight through an XR HMD for instant reference without interrupting the surgery. In summary, XR technology can provide critical information as needed for intraoperative reference and interactive, intuitive remote assistance or discussions. The real-time immersive sharing of holographic 2D/3D medical information in XR technology may open up a new dimension for remote healthcare (50). It contrasts sharply with traditional remote healthcare systems by integrating separate modules for image processing, audio transmission, and video capture, enabling surgeons to go beyond the limitations of cumbersome transmission methods and truly achieve remote connectivity in the surgical environment. This will help create an interconnected 3D digital world in the field of orthopedics, where imaging, videos, 3D models, and other data can be exchanged and made to be mutually beneficial across different locations.

Apart from preoperative planning and intraoperative navigation, AR technology has the potential to be applied in orthopedic surgery training, pain management, and postoperative rehabilitation. AR shows promise in improving pre-surgical and peri-surgical education and training for patients and surgeons. It can also alleviate stress associated with immobilization and reduce movement-induced pain without relying on anxiolytics or analgesics (51, 52).

### 6.2 Disadvantages

Intraoperative navigation requires accurate positioning and relative precision. The registration points should fully cover the surgical space, which is closely related to the reasonable planning and placement of registration points. It is also important to consider the deformation that may occur during the surgical procedure, including that of puncture needles and screws. For patients, due to the use of EM trackers and navigation tools, unusual positions on the operating table may be required (11). Furthermore, as the system accuracy improves to the millimeter level, the small displacements caused by patient breathing also need to be fully considered in order to further enhance the accuracy of navigation systems.

There is still room for improvement in the currently available head-mounted devices on the market. The retail price of Microsoft HoloLens2 is USD 3,500, which is relatively expensive. Although it is more affordable than other navigation systems, HoloLens2 is primarily designed for entertainment and multimedia viewing, and it still lacks accuracy and precision in comparison with others (15–17, 27, 28).

In addition, research has indicated that the contrast of AR models is limited, and the images are affected by bright light. Furthermore, users may experience eye fatigue and visual discomfort during use, highlighting the need for training. At the same time, XR-technology-assisted intraoperative navigation has limited accuracy when applied to patients with special body types, such as obese or deformed patients (53, 54).

Apart from the limitations regarding XR navigation systems themselves, the majority of the surgical applications of XR technology lack proper validation. Although many of the studies cited and discussed in this review showed accuracy and efficacy with the assistance of XR technology, there are other factors (e.g., visualization, user-friendliness, clinical outcomes, and patient recovery) should be taken into consideration to perform a comprehensive validation of XR navigation systems. Moreover, it is also challenging to achieve and demonstrate a statistically significant difference between results with and without the use of an intraoperative XR navigation system. Thus, more studies of the effective and comprehensive evaluation and validation of XR-assisted navigation systems that are employed in surgery are expected (55). Furthermore, one limitation of our study is that surgeries were conducted by experienced orthopedic surgeons in most of the cases in this narrative review, and the high rate of accuracy might have been related to their expertise rather than solely to the XR-technology-assisted navigation systems. This situation is inevitable in most studies, making it difficult to demonstrate an objective difference due solely to the application of XR technology.

## 7 Conclusions

In this review, we examined the current applications of extended reality (XR) technology in orthopedic surgery and objectively evaluated its strengths and limitations. XR-assisted intraoperative navigation technology is widely used in various fields of orthopedic surgery, including trauma, joint, spine, and bone tumor surgery. This navigation technology has unique advantages in intraoperative applications, effectively improving accuracy, reducing complications, shortening surgical duration, and reducing radiation exposure. However, there are still limitations in XR-assisted navigation technology, including the need for improved accuracy, the immaturity of devices, and the lack of standardized specifications and objective evaluation criteria. Further development and refinement are needed in future research and applications.
